# Role of the RAB7 Protein in Tumor Progression and Cisplatin Chemoresistance

**DOI:** 10.3390/cancers11081096

**Published:** 2019-08-01

**Authors:** Flora Guerra, Cecilia Bucci

**Affiliations:** Department of Biological and Environmental Sciences and Technologies (DiSTeBA), University of Salento, Via Provinciale Lecce-Monteroni 165, 73100 Lecce, Italy

**Keywords:** RAB7, endocytosis, tumor progression, cisplatin resistance, extracellular vesicles, oncogene, oncosuppressor

## Abstract

RAB7 is a small guanosine triphosphatase (GTPase) extensively studied as regulator of vesicular trafficking. Indeed, its role is fundamental in several steps of the late endocytic pathway, including endosome maturation, transport from early endosomes to late endosomes and lysosomes, clustering and fusion of late endosomes and lysosomes in the perinuclear region and lysosomal biogenesis. Besides endocytosis, RAB7 is important for a number of other cellular processes among which, autophagy, apoptosis, signaling, and cell migration. Given the importance of RAB7 in these cellular processes, the interest to study the role of RAB7 in cancer progression is widely grown. Here, we describe the current understanding of oncogenic and oncosuppressor functions of RAB7 analyzing cellular context and other environmental factors in which it elicits pro and/or antitumorigenic effects. We also discuss the role of RAB7 in cisplatin resistance associated with its ability to regulate the late endosomal pathway, lysosomal biogenesis and extracellular vesicle secretion. Finally, we examined the potential cancer therapeutic strategies targeting the different molecular events in which RAB7 is involved.

## 1. Introduction

The RAB (Ras-related in brain) protein family comprises small guanosine triphosphatases (GTPases) that are important regulators of membrane identity and of vesicular trafficking events [1,2,3,4]. RAB7A is a small GTPase of the RAB family, ubiquitously expressed, mainly localized to late endosomes and with a pivotal role in endocytic trafficking. Indeed, RAB7A regulates maturation of early endosome into late endosome, transport from early endosomes to late endosome and lysosomes, clustering and fusion of late endosomes and lysosomes in perinuclear region and lysosomal biogenesis [5,6]. In addition of these functions, and related to them, RAB7A has many other cellular roles, being involved in autophagy [7,8], in apoptosis [9], in phagocytosis [10], in retromer regulation, mitophagy, and lipophagy as well as in cytoskeleton organization [5]. RAB7A plays specific key roles in neurons as a regulator of neurotrophin receptor trafficking and signaling, neuritogenesis, axonal retrograde transport, and neuronal migration [5,11,12,13]. Furthermore, RAB7A has been involved in host-pathogen interaction as often microbial or viral pathogens target this protein or its regulators or effector to subvert phagocytosis or other cellular processes [14,15] but also in thyroid hormone production [16] and extracellular vesicle secretion [17].

As all GTPases, RAB7A functions are guaranteed by a cyclical mechanism of activation and inactivation, that depends on GTP binding and hydrolysis, thus shuttling between active GTP-bound and inactive guanosine diphosphate (GDP)-bound state. A number of proteins control this cycle and, among them, the most relevant are the Guanine nucleotide Exchange Factors (GEFs) that stimulate GDP dissociation and GTP acquisition and GTPase-Activating Proteins (GAPs) that are required to prompt GTP hydrolysis. In addition, the Rab Escort Proteins (REPs) recognize newly synthesized RAB7A proteins and present them to the prenylation enzyme Rab geranylgeranyl transferase (RabGGT) allowing geranyl-geranylation, useful for anchorage to membranes. In the cytoplasm, GDP-bound geranyl-geranylated RAB7A is associated to GDP Dissociation Inhibitor (GDI), while GDI displacement is operated by the GDI Displacement Factor (GDF). After GDI displacement, RAB7A is recruited to membranes where it is activated by GEFs through nucleotide exchange [18,19]. Active (GTP-bound) RAB7A is able to interact with several different effector proteins, through which it regulates a number of downstream functions [20]. Two mutants of RAB7 are widely used to study the function of this GTPase: a dominant negative mutant (RAB7^T22N^) characterized by impaired nucleotide exchange, thus locking the protein in its inactive GDP-bound form, and a constitutively active mutant (RAB7^Q67L^) characterized by impaired GTP hydrolysis, thus locking the protein in its active GTP-bound form [5].

Considering the involvement of RAB7A in numerous cellular processes, it is easy to imagine that alterations of its expression, biochemical properties, such as GTP exchange and hydrolysis, interaction with effectors and mutations may cause occurrence of pathological conditions. Indeed, for instance, mutations in the *RAB7A* gene cause the Charcot-Marie-Tooth type 2B (CMT2B) peripheral neuropathy [21,22,23,24,25,26] and there are numerous reports in the literature indicating RAB7A as a lead actor of cancer [5].

The aim of this review is to describe the role of RAB7A in cancer progression and in chemoresistance and to illustrate the emerging and preliminary attempts to target RAB7A and late endocytic pathway as a novel strategy to cancer treatment. 

## 2. RAB7A in Cancer Progression

The tumor pathogenesis is a multistep process characterized by progressive evolvement of normal cells to the neoplastic state acquiring the traits to become tumorigenic and, ultimately, malignant [27,28].

During cancer progression the cells acquire in succession the following capabilities: (i) Sustaining proliferative signaling, (ii) evading growth suppressors, (iii) resisting cell death, (iv) enabling replicative immortality, (v) inducing angiogenesis, (vi) activating invasion and metastasis, (vii) reprogramming of energy metabolism, and (viii) evading immune destruction [29]. Genome instability and inflammation are at the base of these hallmarks. Indeed, acquisition of these hallmarks is expedited by generation of genetic diversity and is promoted by inflammation. Moreover, a further aspect that should not be ignored is that normal cells contribute to the acquisition of the eight hallmarks creating the “tumor microenvironment” [29]. For example, epithelial–mesenchymal transition (EMT) is the process by which cancer cells change their phenotype from epithelial to mesenchymal to become invasive and to start metastatization. This aspect of cancer progression is supported by cancer-associated fibroblasts (CAFs) in the stroma that surrounds the growing tumor mass [29]. 

In the context of cancer progression, genes that determine gain of functions inducing abnormal cellular growth to favor tumorigenesis are known as oncogenes, while tumor suppressors genes protect cells from cancer progression until loss of heterozygosis (LOH) [30]. According to these definitions RAB7A was described as oncogene or tumor suppressor depending of its capacity to favor tumor progression or to prevent malignant growth, inducing or blocking different aspects within the eight hallmarks of cancer. The different impact of RAB7A in cancer progression, probably depending on the cellular context and on other environmental factors, is analyzed below. 

### 2.1. Oncogenic Functions of RAB7A

EMT is characterized by several molecular changes, including downregulation of epithelial markers and key components of intercellular junctions, such as E-cadherin, claudins, and occludins, disruption of desmosomes as well as upregulation of the mesenchymal markers N-cadherin, vimentin and fibronectin, thus fostering motility and invasion. EMT allows the cells to gain migratory and invasive features through degradation of extracellular matrix (ECM) proteins, actin cytoskeleton reorganization, formation of membrane protrusions required for invasive growth, extravasation, angiogenesis, as well as anoikis evasion and drug resistance [31,32].

Recently, it was discovered that RAB7A is important for actin cytoskeleton organization [33,34,35]. Indeed, RAB7 interacts with RAC1, a small GTPase involved in the regulation of actin cytoskeleton [36]. Overexpression of RAB7 increases RAC1 activity, while RAB7 silencing caused RAC1 inactivation [36]. Moreover, ARMUS, an effector of RAC1, is a TBC RAB-GAP that inactivates RAB7 coordinating RAB7 and RAC1 functions during autophagy [34,35].

Among several RAB7A effectors, there are two members of the intermediate filament (IFs) proteins, vimentin, and peripherin. IFs, one of the three components of cytoskeleton, are constituted through the assembly of soluble precursors into insoluble protein polymers [37,38]. IFs not only are the major determinants of cell architecture, but also regulation of membrane trafficking and organelle positioning and function dependent on them [39]. Furthermore, it is known that upregulation of vimentin in epithelial cells favors acquisition of the mesenchymal phenotype increasing cell motility, inducing physical changes in cell shape, loss of cell–cell contacts, and increasing the turnover of focal adhesions [40,41]. Coherently, loss of vimentin in mesenchymal MDA-MB-231 cells significantly decreases the ability of these cells to migrate and invade [42]. Furthermore, RAB7A is able to regulate cell migration through Ras-related C3 botulinum toxin substrate 1 (RAC1) and vimentin in human lung cancer NCI H1299 cells [43]. Direct interaction between vimentin and RAB7A allows RAB7A-mediated regulation of vimentin filament assembly [44]. Indeed, vimentin phosphorylation is increased by overexpression of RAB7A^wt^ or of the constitutively active RAB7A^Q67L^ mutant, with consequent redistribution of vimentin in the soluble fraction, while decreased of vimentin phosphorylation and increased of amount of filamentous vimentin was determined by RAB7A depletion [44]. Moreover, reorientation of vimentin during migration is determined by RAB7A [43]. RAB7A depletion strongly affects cell velocity and directness during migration, determining alteration of β1-integrin activation, distribution, and trafficking and thus hampering cell adhesion and spreading [43]. Furthermore, RAB7A depletion reduces RAC1 activation, interfering with filopodia formation and, altering vimentin organization [43]. 

Tumor-necrosis factor-α (TNF-α) and TNF-β are among stimuli required to induce EMT [45]. After stimulation with TNF-α to induce EMT and invasiveness, RAB7A expression was increased in cholangiocarcinoma cells [45]. Also in thyroid adenomas and in ovarian/primary peritoneal serous carcinoma overexpression of RAB7A was observed in cancerous tissues [16,46].

In highly aggressive tumors, such as ovarian, melanoma, breast and colorectal the capacity of primary tumor cells to invade the surrounding tissues through ECM degradation is linked to the expression of matrix metalloproteinases (MMPs) [47,48,49,50,51]. Furthermore, membrane-anchored membrane type 1 matrix metalloproteinase (MT1-MMP) has a central role in this process [49,52,53,54]. To this purpose, MT1-MMP induces degradation of ECM through its proteolytic function determining cleavage of collagen types I, II, and III, laminins 1 and 5, fibronectin, vitronectin, fibrin, and aggrecan, but also it is able to activate pro-MMPs [55]. Once activated, MT1-MMP is transported to the plasma membrane in order to mediate ECM proteolytic degradation and to favor cell invasion. Proteolytic activity of MT1-MMP is reduced by internalization [56,57], but internalization causes also recycling of MT1-MMP back to the plasma membrane and therefore it is essential for MT1-MMP degradative function [58]. It has been demonstrated in cervical carcinoma HeLa and in fibrosarcoma HT-1080 cells that endosomal trafficking and recycling of MT1-MMP1 are dependent on RAB7A and VAMP7 [59]. Interestingly, inhibition of recycling, using dominant negative mutants of RAB7 and VAMP7, reduces both invasion and migration [59]. Thus, it has been demonstrated a role of RAB7A in the recycling pathway, which is important for RAB7A oncogenic functions [59]. 

Epidermal Growth Factor Receptor (EGFR) and Human Epidermal growth factor Receptor 2 (HER2) are members of receptor tyrosine kinases (RTKs) ErbB family. High expression of both proteins is detected in breast cancer and it is associated with more aggressive cancer phenotypes and poorer prognosis [60]. Indeed, RTKs are activated by ligand binding with subsequent dimerization, internalization and initiation of pro-oncogenic intracellular signaling via mitogen-activated protein kinase (MAPK) and PI3K/Akt pathways. Thus, enhanced expression/activation of EGFR or HER2 determines aberrant expression of genes involved in cancer proliferation, survival, migration, and angiogenesis [61]. Endocytosis-mediated internalization of ligand-bound RTKs is an important process for the regulation of downstream signaling [62]. Indeed, activated receptors, after internalization, are sorted in early endosomes and subsequently recycled to the plasma membrane or degraded [63]. The recycling of receptors allows maintenance of activated receptor number on plasma membrane and is responsible for prolongated signaling [63]. RAB7 is essential for the degradation of signaling receptors, being responsible of sorting them into late endosome and of their transport to lysosomes [64]. Indeed, for instance, in HeLa cells the kinetic of EGFR degradation is accelerated or slowed by expressing the constitutively active or the dominant negative mutant of RAB7, respectively [65]. Intriguingly, it was demonstrated that, in human cervix squamous carcinoma A431 and breast cancer MCF7 cells, RAB7 is responsible for Akt survival signal maintenance during cell detachment or when HSP90 is inhibited, protecting cells from apoptosis [66]. In fact, RAB7 silencing suppresses anchorage-independent growth with a concomitant increase of *anoikis* and a reduction of active phosphorylated Akt [66]. In agreement, in breast cancer cells, the RAB7 interactor Rabring 7 was discovered to have a key role [67,68]. Accordingly, the gene was renamed Breast Cancer Associated gene 2 (BCA2) [67,68]. Overexpression of BCA2 in HeLa cells causes failure of RAB7 membrane association and thus RAB7 remains in the cytosol, not being able to carry out its functions on the endosomal membranes [69]. As a consequence, EGFR degradation is inhibited [69].

Phosphatase and tensin homologue (PTEN) is an important tumor suppressor, mutated in various types of cancers [70]. As a dual specific phosphatase, PTEN acts on both lipid and protein substrates being responsible for suppression of EGFR-mediated cell growth and proliferation signaling [71,72]. Moreover, phosphatidylinositol-3,4,5-trisphosphate (PIP3) is converted into phosphatidylinositol-4,5-bisphosphate (PIP2) at the cellular membrane by PTEN, which also negatively regulates oncogenic PI3K-AKT signaling and it is associated with PI(3)P-containing endosomes [73]. RAB7 S72 and Y183 residues are essential for maturation of late endosomes. Indeed, both residues guarantee RAB7-GDI association, subsequent delivery to late endosomal membranes and activation by the Mon1a–Ccz1 GEF complex [74,75]. Recent work has discovered in HeLa and in human embryonic kidney HEK 293T cells a PTEN-dependent regulation of RAB7, strengthening the link between RAB7 and cancer [74]. PTEN controls EGFR signaling through activation of RAB7-mediated endosome maturation, acting on S72 and Y183 residues of RAB7 [74]. Mutations of *PTEN* are very frequent in several tumors [76,77]. Notably, the authors demonstrated that PTEN mutation at residue 138, determining oncosuppressor inactivation, affects RAB7 dephosphorylation at S72 and Y183 residues, with loss of the control of RAB7-dependent endosomal degradation and consequent uninterrupted growth signaling with important implications for tumor progression [74]. 

Cancer proliferation can arise also when immune surveillance is compromised and unchecked tumor cells proliferate and grow to form tumor [78]. Indeed, inflammatory response has a pivotal role in different stages of cancer progression, including initiation, promotion, malignant phenotype acquisition, invasion and metastasis [79,80]. Myeloid-derived suppressor cells (MDSCs) expansion is one of manifestations of inflammations [81]. MDSCs are able to suppress immune surveillance and directly induce tumor cell proliferation in vitro, and tumor growth and invasion in vivo [82,83,84,85]. Metabolic reprogramming and evading immune destruction are two of cancer important hallmarks. Interestingly, metabolic reprogramming of MDSCs is regulated by Lysosomal Acid Lipase (LAL). LAL generates free cholesterol and free fatty acids in the lysosomes through hydrolysis of cholesteryl esters and triglycerides [84]. LAL deficient (*lal*^−/−)^ MDSCs compensate energetic deficit through increase glycolytic metabolism, ATP production, reactive oxygen species (ROS) over-production and overactivation of mammalian target of rapamycin (mTOR) [84]. In *lal*^−/−^ MDSCs, development, systemic expansion, trans-endothelial migration, immune suppression and direct stimulation of tumor cell proliferation are regulated by mTOR [80,82,83,84,86,87]. RAB7 interacts with mTOR and is upregulated in *lal*^−/−^ MDSCs [80,88]. A direct role of RAB7 in the regulation of MDSCs metabolism was demonstrated [80,88]. In fact, depletion of RAB7A in *lal*^−/−^ MDSCs reduced overactivation of mTOR, decreased glucose consumption and ROS over-production, and increased the number of healthy mitochondria [80,88]. Moreover, it was demonstrated that RAB7 knockdown reduced *lal*^−/−^ MDSCs differentiation from bone marrow, decreased trans-endothelial migration and abrogated T cell suppression [88]. Furthermore, RAB7 knockdown reduced the ability of MDSCs to induce tumor cell proliferation in vitro, tumor growth and tumor invasion in vivo [88]. Similar to what was observed in *lal*^−/−^ MDSCs, RAB7 is upregulated also in *lal*^−/−^ endothelial cells (ECs) [89]. Also, stimulation of RAB7 activity determines the ability of *lal*^−/−^ ECs to stimulate tumor cell proliferation and metastasis [89]. Importantly, inhibition of RAB7 in *lal*^−/−^ ECs was able to revert the tumor phenotype causing reduction of their enhanced migration and suppressing in vitro proliferation and in vivo tumor growth and metastasis [89]. 

**Figure 1:**
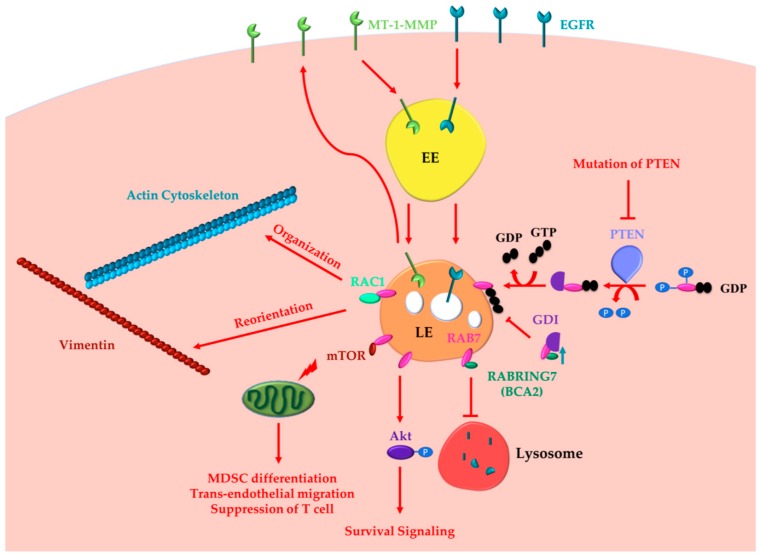
RAB7 (Ras-related in brain 7) oncogenic functions in cancer progression. RAB7A determines the acquisition of invasion and metastasis features through different routes: (i) inducing actin cytoskeleton organization and vimentin reorientation thanks to RAC1 (Ras-related C3 botulinum toxin substrate 1) interaction; (ii) determining internalization and recycling of MT-1-MMP to allow extracellular matrix degradation. Other cancer hallmarks, such as metabolic reprogramming, evasion from immune destruction and angiogenesis, are induced by RAB7 through interaction with mTOR (mammalian target of rapamycin). Indeed, RAB7 induces activation of mammalian target of rapamycin (mTOR) decreasing the number of healthy mitochondria and inducing MDSC (myeloid-derived suppressor cells) differentiation, trans-endothelial migration, and T cell suppression. RAB7 induces Akt-mediated survival signaling. RAB7 interacts with Rabring7, also named Breast Cancer Associated gene 2 (BCA2), and inhibits Epidermal Growth Factor Receptor (EGFR) degradation, while BCA2-overexpression determines RAB7 sequestration in the cytosol by the GDP (guanosine diphosphate) Dissociation Inhibitor (GDI), and prevents EGFR degradation. Also, *PTEN* (phosphatase and tensin homologue) mutations, determining lack of RAB7 dephosphorylation and impairment of endosomal maturation, inhibit EGFR degradation. These latter oncogenic functions of RAB7 are involved in the acquisition of three cancer hallmarks: resisting cell death, evading growth suppressors and sustained proliferative signaling. EE, Early Endosome; LE, Late Endosome.

Altogether these data demonstrate the oncogenic functions of RAB7 (Figure 1).

### 2.2. Oncosuppressor Functions of RAB7A

An important obstacle to neoplastic transformation is represented by growth factor dependence. Indeed, lack of growth factors induces withdrawal of cells from the cell cycle and activation of apoptosis. Growth factors induce cell proliferation but also suppression of intrinsic mitochondria-mediated apoptosis [90]. In fact, deprivation of growth factors induces rapidly a decline of the rate of glucose and amino acid uptake, and loss of the receptors responsible for the cellular uptake of iron (transferrin receptor) and cholesterol (LDL receptor) from the surface of the cells [91,92,93,94]. These metabolic alterations induce destabilization of mitochondrial physiology and bioenergetic dysfunction with consequent decrease of mitochondrial membrane potential and final release of proapoptotic mediators [95,96].

Tumor cells may acquire advantageous mutations to gain growth factor independence, to avoid apoptosis and to proliferate in the absence of extrinsic signals. Growth factor independent survival is supported by the activated form of the oncogene AKT, which maintains high rate of glycolysis, preserves mitochondrial membrane potential and prevents apoptosis [91]. It was demonstrated that RAB7 has a role in growth factor independent survival [91]. In fact, degradation of nutrient transporters is prevented by inhibition of RAB7 despite growth factor withdrawal [91]. Indeed, in murine prolymphocytic FL5.12 RAB7-depleted cells, proteins that have entered the endocytic pathway and that normally would be degraded in lysosomes, are instead recycled to the plasma membrane and re-expressed on the cell surface [91]. Thus, during growth factor withdrawal expression of the dominant negative mutant RAB7^T22N^ causes maintenance of the mitochondrial membrane potential [90]. Also, in TP53 (Tumor Protein p53) null primary mouse embryonic fibroblasts, expression of the RAB7^T22N^ dominant negative mutant cooperates with the adenoviral protein E1A, which inhibits Rb (retinoblastoma tumor suppressor), in order to promote cancer transformation [90]. Accordingly, it was established that RAB7 activity is regulated by growth factor availability in murine prolymphocytic FL5.12 cells [97]. In fact, during nutrient deprivation RAB7 moves from the cytosol to the late endosomal and lysosomal membranes with a consequent consistent increase of the GTP-bound form [97]. Moreover, expression of the constitutively active RAB7^Q67L^ mutant, which displays impaired GTPase activity and is mostly GTP-bound, is sufficient to trigger apoptosis even in the presence of growth factors and it is also able to reverse independent growth factor survival induced by protein kinase C (PKC) δ inhibition [97]. 

Oncosuppressor-like functions of RAB7 have also been described in prostate cancer [98]. It is known that in human prostate cancer DU-145 cells the intracellular localization of lysosomes generally closer to the cell surface determines the secretion of proteases favoring cell invasion [98,99]. Instead, perinuclear localization of lysosomes is a common feature of less invasive cells, which do not usually secrete large amounts of acid hydrolases. The movement of lysosomes in the cells are guaranteed by microtubules and actin filaments utilizing molecular motor protein such as dynein, kinesin, and/or myosin family members [100]. In addition, several GTPases, among which RAB7, regulate motor protein activity determining the intracellular distribution of lysosomes and other organelles in the cell [5]. The reported RAB7 role as a tumor suppressor is based on the fact that it is a negative regulator of prostate cancer growth and invasion because it inhibits ligand-induced c-Met signaling, known to induce EMT, and it controls the perinuclear localization of lysosomes [98]. Indeed, depletion of RAB7 strongly increases secretion of proteases, supporting larger tumors formation in vivo and increased invasive capability into surrounding tissue [98]. Troglitazone is an agonist of the peroxisome proliferator-activated receptor-γ (PPAR-γ), used for the treatment of type II diabetes because of its ability to improve insulin sensitivity [101]. Interestingly, this compound has several PPAR-γ-independent effects and, for instance, influences cell migration and invasion in several malignancies [102]. Indeed, Troglitazone and other members of the Thiazolinedione family inhibit RAB7-induced lysosome trafficking towards the cell surface and consequent Cathepsin B secretion [98].

The RAB7 behavior as tumor suppressor has been demonstrated also in glioblastoma [103]. Extensive activation of RTKs was found in greater than 45% of gliobastoma specimens harboring EGFR amplification or mutations [104]. CD44 is a cell surface receptor responsible for increasing RTKs signaling and defined also as co-receptor. CD44 is characterized by extensive alternative splicing that produces two main families of isoforms, termed CD44v and CD44s [105]. The role of the CD44s splice isoform has been studied and it was discovered that it is involved in attenuation of endocytosis-mediated EGFR degradation and in sustaining downstream Akt signaling [103]. Interestingly, CD44s interacts with RAB7A and blocks RAB7-mediated EGFR degradation by stimulating GTP hydrolysis in glioblastoma multiform cell lines [103].

Several RAB7-GAP have been identified and among them there is the phospho-protein folliculin (FLCN) [106]. Mutations of the *FLCN* gene are the cause of Birt–Hogg–Dubé disease characterized by the development of renal cell cancer, benign skin lesions, and lung cystis [107]. RAB7 interacts with FLCN and together they are able to regulate EGFR signaling [106]. Indeed, in FLCN-deficient thyroid cancer FTC-133 cells, dysregulation of RAB7 activity due to loss of FLCN determined slower EGFR degradation, increased EGFR signaling, and tumor growth [106].

Finally, RAB7 is activated by Liver Kinase B1 (LKB1), a serine–threonine protein kinase of calcium calmodulin family, ubiquitously expressed in several tissues, including liver, heart, lung, and skeletal muscle [108]. Hypoxia determines LKB1 nuclear export to the cytosol where it interacts with the Neuropilin-1 (NRP-1) protein and with RAB7 [108]. NRP-1 is a receptor of the Vascular Endothelial Growth Factor (VEGF) and its enhanced expression during tumorigenesis determines the angiogenetic switch with a role in cell survival, migration and invasion [109,110,111]. LKB1 binds to the constitutively active RAB7^Q67L^ mutant protein but not to the dominant negative RAB7^T22N^ mutant protein, indicating that it binds preferentially to the GTP-bound form of RAB7 [108]. LKB1 has been identified in lung cancer A549 cell lines as a RAB7 effector that alters NRP-1 trafficking causing NRP-1 localization to lysosomes and subsequent NRP-1 degradation [108]. The lysosomal degradation of NRP-1 causes decreased tumor angiogenesis and decreased tumor growth in vivo [108]. In contrast, depletion of RAB7 by RNA interference blocks transfer of NRP-1 to lysosomes, causing increased NRP-1 expression, increased angiogenesis and increased tumor growth [108].

**Figure 2:**
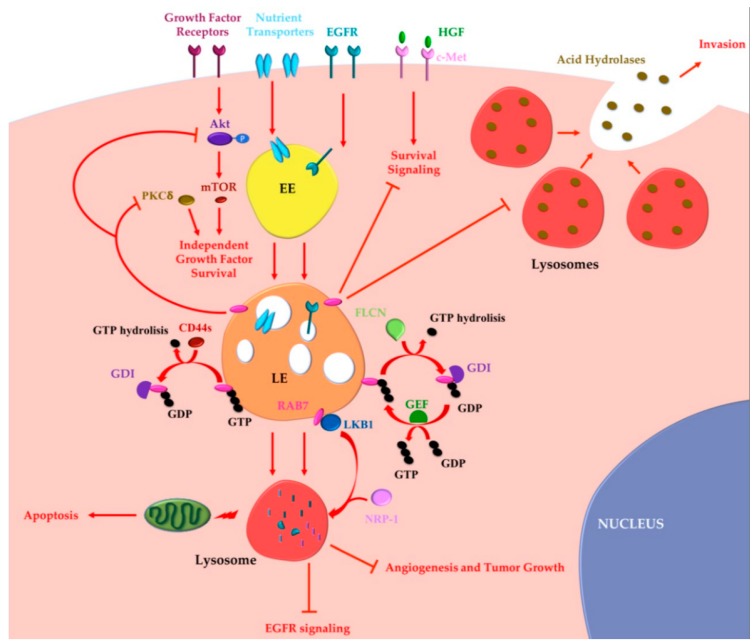
RAB7 oncosuppressor functions in cancer progression. Tumor cells may acquire growth factor independence in order to avoid apoptosis and to proliferate in the absence of extrinsic signals. RAB7 is able to reverse independent growth factor through inhibition of Akt and PKCδ-regulated survival signaling. Moreover, during growth factor withdrawal, nutrient transporters are removed from the cell surface and degraded by lysosomes in a RAB7-regulated process. Nutrient transporters degradation determines impairment of cellular bioenergetics, loss of mitochondrial homeostasis and, finally, activation of apoptosis preventing growth factor-independent tumor proliferation. RAB7 is essential for EGFR degradation and for removal of sustained proliferative signals. EGFR degradation is also guaranteed by GAP (GTPase-Activating Protein) FLCN (folliculin) activity, which allows cyclical RAB7 regulation. CD44s inhibits RAB7 by inducing GTP hydrolysis and attenuation of endocytosis-mediated EGFR degradation, underling the importance of RAB7 in EGFR degradation. Liver Kinase B1 (LKB1) is an effector of RAB7, which induces Neuropilin-1 (NRP-1) lysosomal localization and degradation with consequent decreased tumor angiogenesis and tumor growth. RAB7 is also a negative regulator of the Hepatocyte Growth Factor (HGF)-Met signaling axis and controls the perinuclear localization of lysosomes avoiding lysosomal peripheral localization, acid hydrolases secretion and cancer invasion. EE, Early Endosome; LE, Late Endosome; GEF, GTP Exchange Factor; GDI, GDP Dissociation Inhibitor.

Altogether these data indicate that RAB7 can also exert oncosuppressor functions (Figure 2).

### 2.3. Is RAB7A an Oncojanus?

Gasparre and coworkers have coined the name *oncojanus* to identify a novel-type of tumor-implicated genes that have the ability to behave either as oncogenes or oncosuppressor during tumorigenesis [112]. In fact, they discovered that for a number of mitochondrial genes of which both oncogenic and suppressor roles have been demonstrated, tumor growth arrest is induced when disruptive mutations reach a critical mutant load [112,113]. In contrast, nondisruptive or below threshold mutations in the same genes prompt tumor growth [112,113]. 

Interestingly, also the *RAB7A* gene can behave as an oncojanus with oncogenic or oncosuppressor roles. Indeed, expression levels of RAB7 change during melanoma tumor progression [114]. Initially, in benign nevi, RAB7 is expressed at low levels under regulation of the transcription factor Sex determining region Y (SRY)-related High Motility Group (HMG)-box 10 (SOX10) [114]. Then, in primary non-invasive melanomas, RAB7 expression is strongly increased and under the control of the MYC oncogene [114]. These observations have suggested role of RAB7 in benign nevi transformation into melanoma. However, in primary invasive melanomas, RAB7 expression is strongly reduced, although never abolished, compared to non invasive ones [114]. Thus, RAB7 downregulation at this stage seems to important for acquisition of invasive properties [114]. In addition, downregulation of RAB7 correlates with increasing risk of development of metastasis [114]. Finally, in melanoma metastasis expression of RAB7 seems to increase compared to primary invasive melanomas [114]. Thus, RAB7 expression varies during tumor progression suggesting that in the first step of transformation from benign nevi to non-invasive melanoma RAB7 behaves as an oncogene while in the following progression to invasive melanoma it could as a suppressor and therefore is downregulated.

RAB7 seems to behave as an oncojanus also in inflammatory breast cancer. Inflammatory breast cancer is a lethal form of breast cancer characterized by excessive lymphovascular invasion, which is responsible of metastatic dissemination, resistance to chemotherapy and cancer recurrence [115]. MARY-X is a human xenograft model of inflammatory breast cancer representing a good tool to investigate this kind of cancer as it forms spheroids that have biological similarities with the lymphovascular embolus but also with the blastocyst [116]. In the lymphovascular embolus, active clonal selection in the main tumor mass takes place and the stem cell phenotype emerges, exhibiting enhanced stem cell signaling and survival pathways [117,118]. Only a fraction of MARY-X become lymphovascular emboli in vivo and only a fraction of MARY-X cells forms spheroids in vitro. Nevertheless, sequential events of MARY-X spheroidogenesis in vitro have been characterized and include modulation of RAB7 expression and RAB7-regulated production of different forms of E-cadherin [119]. Besides the fact that modulation of RAB7 expression by silencing or overexpression alter spheroidogenesis events, it is interesting to note that the first steps of spheroidogenesis, the early aggregate-to-spheroid transition stages, were characterized by downregulation of RAB7 expression while the well-formed compact spheroids were characterized by strong RAB7 expression [119]. Therefore, also in this case, RAB7 expression follows a complex pattern as initially down-regulation of RAB7 is required for the formation of the loose spheroids but then high RAB7 expression is required to form compact and well-formed spheroids [119], suggesting that RAB7 behaves as an oncojanus.

## 3. Role of RAB7 in Cisplatin Chemoresistance Mediated by Extracellular Vesicles

Cisplatin is a well-known platinum coordination compound currently used as a drug in the therapy of different types of cancer, such as ovary, testicular, head and neck, bladder, and lung cancer. Unfortunately, intrinsic and acquired chemoresistance to cisplatin render often the therapy unsuccessful for patients and the multifactorial and redundant nature of this phenomenon poses a significant barrier against the identification of effective chemosensitization strategies.

Recent research confers to RAB7 a specific role in the mechanism of chemoresistance to cisplatin. Hypoxia is a critical condition, which favor the spread of epithelial cancers [120]. The hypoxic microenvironment promotes the release of exosomes determining the acquisition of a more aggressive phenotype and resistance to cisplatin [121]. Exosomes are defined as extracellular vesicles (EVs) of endosomal origin with a diameter of 50–150 nm. Their precursors are intraluminal vesicles (ILVs), which are originated by inward budding of domains of the early endosomal membranes and which are present in multivesicular bodies (MVBs). MVBs are a station of endocytosis between early endosomes and late endosomes and material present in MVBs is mainly destined to lysosomes to be degraded. However, in particular conditions, MVBs move towards the plasma membrane and fuse with the plasma membrane, releasing their ILVs in the extracellular space [122]. These extracellular vesicles were defined exosomes [122]. Recently, it was observed that, in hypoxic conditions, ovarian cancer cells, through the action of the oncogenic transcription factor Signal Transducer and Activator of Transcription 3 (STAT3,) upregulate RAB27 (a RAB protein controlling late endosome docking with the plasma membrane) and downregulate RAB7 [121]. Analyzing the concentration of exosomes in sera from cisplatin-resistant and sensitive ovarian cancer patients, the authors found a significant increase in the number of exosomes released in sera of cisplatin-resistant compared to drug sensitive patients [121]. In addition, the analysis of cisplatin in exosomes has revelated that hypoxia impacts on the exosomal-mediated efflux of cisplatin from ovarian cancer cells [121]. Interestingly, incubation of cells with exosomes derived from hypoxic ovarian cancer cells induces resistance to platinum compounds [121]. Moreover, the release of EV as a novel mechanism of chemoresistance has been described in ovarian cancer [123].

**Figure 3:**
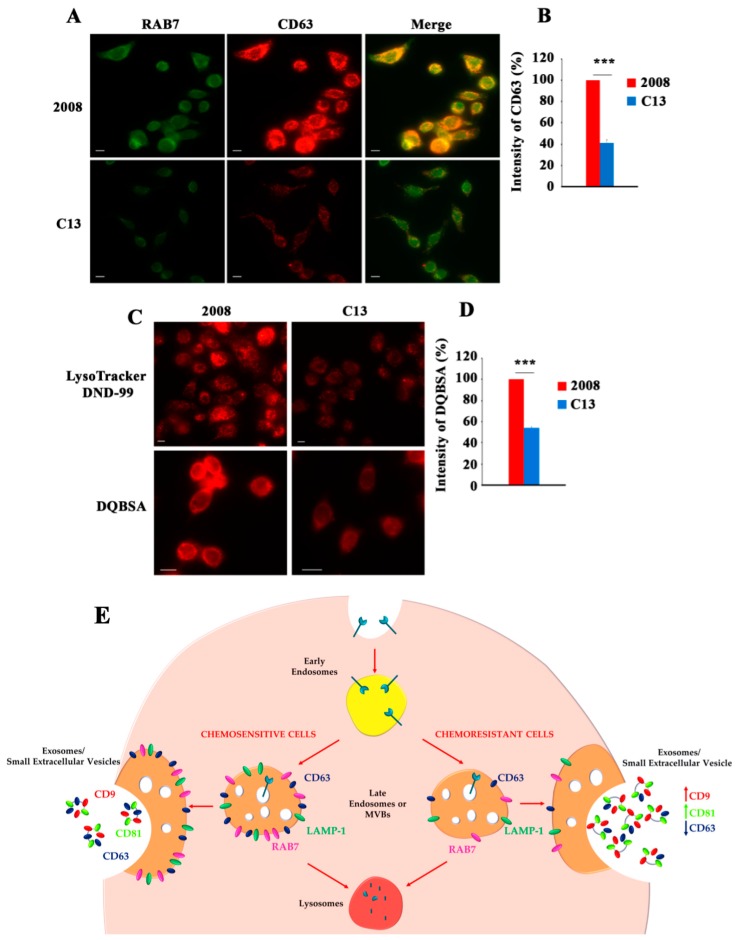
RAB7 regulates extracellular vesicle secretion in cisplatin resistant cells. (**A**) 2008 and C13 cervical cancer cells were immunostained with anti-RAB7 (green) and anti-CD63 antibodies (red) Scale bar: 10 μm. (**B**) Immunofluorescence intensity of CD63 was quantified by ImageJ software. (**C**) Cells were labeled live with Lysotracker Red DND-99 (red) or incubated in the presence of DQ-Red BSA as indicated. Scale bar: 10 μm (**D**) Fluorescence intensity of DQ-Red BSA was quantified by ImageJ software (Version 1.5Oi, Bethesda, MD, USA). Data represent the mean ± standard error of at least three independent experiments (*** *p* ≤ 0.001). (**E**) Intraluminal vesicles (ILVs) of multivesicular bodies (MVBs) and late endosomes are precursors of exosomes/small extracellular vesicles (sEVs). ILVs are generated by the inward budding of discrete domains of the membrane of early endosomes, which subsequently mature into MVBs and late endosomes. MVBs can be directed to lysosomes or to plasma membrane (PM). Fusion of MVBs with PM determines exosomes/sEVs release in the extracellular space. RAB7, LAMP-1 and CD63 are localized onto late endosomes/MVBs and they are downregulated in chemoresistant cells. RAB7 downregulation causes chemoresistance by increasing release of CD9- and CD81-positive exosomes. MVBs, Multivesicular Bodies.

We also have demonstrated that RAB7 plays a key role in cisplatin chemoresistance [17]. Indeed, we found downregulation of RAB7 expression in a number of cisplatin resistant cell lines compared to their sensitive counterparts [17]. This downregulation is shown in Figure 3A for cervical cancer cell line 2008 and its chemoresistant subclone C13. Indeed, immunofluorescence analysis of RAB7 shows a strong perinuclear vesicular staining for chemosensitive 2008 cells (Figure 3A). 

In contrast, C13 cells, the chemoresistant counterpart of 2008 cells, show a much weaker staining, indicating a downregulation of RAB7 (Figure 3A). We have previously demonstrated that C13 chemoresistant cells are characterized by downregulation of RAB7, LAMP1 (Lysosomal associated membrane protein I), and V1G1 (the G1 subunit of the V1 domain of the vacuolar ATPase) [17]. According to these data also CD63 (known as LAMP3) is strongly decreased in chemoresistant C13 cells compared to their sensitive counterpart (Figure 3A,B) suggesting that the entire endolysosomal pathway is affected by RAB7 downregulation. Indeed, CD63 staining is reduced of about 60% in C13 cells (Figure 3B). C13 chemoresistant cells are also characterized by a much weaker staining with Lysotracker DND-99, a probe that labels acidic lysosomes, compared to chemosensitive cells, confirming previous data [17] and suggesting that chemoresistant cells have less acidic compartments (Figure 3B). A decrease in the amount of acidic lysosomes let us to hypothesize that in these cells lysosomal activity was reduced and we used DQ-Red BSA to investigate this issue. Degradation of DQ-Red BSA in lysosomes causes dequenching and thus presence of fluorescence in cells can be used as a measure of lysosomal activity. Interestingly, DQ-Red BSA assay in 2008 and C13 cells revealed a very weak staining in C13 chemoresistant cells, demonstrating lower lysosomal activity compared to 2008 chemosensitive cells (Figure 3C). Quantification of the staining revealed a reduction of about 50% (Figure 3D).

Importantly, depletion of RAB7 by RNA interference induced the resistant phenotype in cisplatin sensitive cells, while overexpression of RAB7 determined sensitization of cisplatin-resistant cells, demonstrating a direct role of RAB7 in cisplatin chemoresistance [17]. Interestingly, we also found that RAB7-mediated chemoresistance is associated with lower intracellular amount of cisplatin [17]. Purification and analysis of small extracellular vesicles (EVs) from chemoresistant and chemosensitive cells revealed increased secretion of CD9- and CD81-positive EVs from chemoresistant cells, indicating that the intracellular reduction of cisplatin could be due to increased efflux through EVs [17]. Notably, modulation of RAB7 expression influenced secretion of CD9- and CD81-positive EVs, suggesting that RAB7 is important for EV secretion and impacting on the cisplatin resistance mechanism [17]. Thus, EVs are important not only as biomarkers but also because they are involved in chemoresistance (Figure 3E) [124].

Altogether, these data indicate that RAB7, because of its role in regulating the late endocytic pathway and EV secretion, controls cisplatin chemoresistance in cervical cancer cells.

## 4. Targeting of RAB7 as Novel Strategy for Cancer Treatment

In light of the emerging role of the endosomal and lysosomal pathway in cancer progression and in response to chemotherapy, it is not difficult to imagine that novel therapeutical strategies could contemplate also late endocytic compartments and factors as targets for cancer treatment. In fact, many reports indicate that, for instance, inhibition of autophagy, a pathway regulated by a number of endocytic proteins, improves anticancer drug resistance of cancer cells [125,126]. Chloroquine (CQ) and hydroxychloroquine (HQ) are currently clinically used in cancer therapy [127]. 

Cancer Stem Cells (CSCs) represent the population of cells in a tumor that are important for the formation of cancer tissue, its maintenance and spreading [128]. CSCs are responsible also for cancer heterogeneity and acquisition of chemoresistance [129]. Thus, CSCs are becoming the new targets for an effective cancer therapy [130]. In a recent work it was demonstrated that RAB5 and RAB7 are important for CSC survival acting on the mitophagy pathway [131]. Importantly, RAB5 and RAB7 inhibition eliminated colorectal CSCs and disrupted cancer foci [131]. The authors also showed that a new compound, the antimalarial drug mefloquine hydrochloride (MQ), inhibits lysosomal activity by specifically reducing expression of RAB5, RAB7, and LAMP proteins [131]. Thus, the authors proposed the use of this drug in combination with classical chemotherapeutical molecules to fight cancer [131].

The crosstalk between mitochondria and lysosomes has gained attention from a growing number of researchers [132,133,134,135,136]. Actually, MQ affects not only the endosomal and lysosomal pathway but also mitochondria as it works suppressing PINK1/PARKIN-mediated mitophagy and inducing mitochondrial dysfunction with the final effect of inducing apoptosis. Therefore, MQ, through these mechanisms of action causes depletion of CSCs and thus, when used as adjuvant of anticancer drugs, demolishes cancer hierarchy [131].

Triple negative breast cancer (TNBC) lacks targeted therapy and its prognosis is the poorest overall compared to other cancer types [137]. In recent clinical trials histone deacetylase inhibitors, such as Vorinostat, have been used for therapy of TNBC [138]. Vorinostat has a moderate action on TNBC cells, inducing apoptosis and arresting cell cycle in cancer cells [139,140]. Because cancer cells upregulate autophagy to favor resistance to drug treatment [141] and Vorinostat, unfortunately, is an inductor of autophagy, recent work proposed the synergistic use of Simvastatin and Vorinostat [142]. Indeed, Simvastatin, an inhibitor of 3-hydroxy-3-methyl-glutaryl-CoenzymeA (HMG-CoA) reductase, which converts HMG-CoA into mevalonate, the limiting step in the cholesterol biosynthetic pathway, strengthens the pro-apoptotic effect of Vorostatin on TNBC through the inhibition of RAB7 prenylation [143]. As a consequence, given the role of RAB7 in autophagosome-lysosome fusion, the biogenesis of autolysosomes and the autophagic flux are inhibited [143]. The role of N6-isopentenyladenosine (iPA), a product of the mevalonate pathway was also elucidated [144]. This compound induces impairment of the autophagic flux through AMPK activation and inhibition of RAB7 prenylation [144]. Importantly, the authors demonstrated that this compound determined the arrest of proliferation of melanoma cancer cells in vitro and the inhibition of tumor growth in vivo [144].

Altogether these data indicate that targeting RAB7 could be useful to identify effective cancer therapy.

## 5. Conclusions

From the discussed data it is clear that RAB7 has important roles in cancer progression, with both oncogenic and oncosuppressor behavior, depending on the cellular context and on environmental factors. Also, it has been recently discovered that RAB7 plays a key role in cisplatin chemoresistance due to its ability of regulating the late endosomal pathway, lysosomal biogenesis, and EV secretion. Given these important roles of RAB7 in cancer progression and in chemotherapy response, it stands to reason that targeting of RAB7 and of RAB7 related pathways could benefit cancer therapy. Clearly, more work is necessary to investigate at the molecular level all the mechanisms involving RAB7. However, the importance of RAB7 targeting or of targeting of RAB7-related pathways, such as the endocytic and/or the autophagy pathway, could be useful not only for cancer but also for a plethora of other diseases, including neurodegenerative and infectious diseases. Indeed, modulation of RAB7 expression and/or activity, considering the multiple key roles of this GTPase in membrane traffic and, in particular, in lysosomal biogenesis, activity and exocytosis, affects cellular homeostasis and could help to prevent and control, for instance, Parkinson’s and Alzheimer’s diseases and, in general, neurological disorders with alterations in RAB7-dependent membrane trafficking events.
